# Complexity synchronization: a measure of interaction between the brain, heart and lungs

**DOI:** 10.1038/s41598-023-38622-8

**Published:** 2023-07-15

**Authors:** Korosh Mahmoodi, Scott E. Kerick, Paolo Grigolini, Piotr J. Franaszczuk, Bruce J. West

**Affiliations:** 1 US Combat Capabilities Command, Army Research Laboratory, Aberdeen Proving Ground, MD 21005 USA; 2 Center for Nonlinear Science, University of North Texas, P.O. Box 311427, Denton, TX 76203 USA; 3 Department of Neurology, Johns Hopkins University School of Medicine, Baltimore, MD 21287 USA; 4 Office of Research and Innovation, North Carolina State University, Raleigh, NC 27695 USA

**Keywords:** Complexity, Dynamical systems, Nonlinear dynamics, Oscillators, Time series

## Abstract

Herein we address the measurable consequences of the network effect (NE) on time series generated by different parts of the brain, heart, and lung organ-networks (ONs), which are directly related to their inter-network and intra-network interactions. Moreover, these same physiologic ONs have been shown to generate crucial event (CE) time series, and herein are shown, using modified diffusion entropy analysis (MDEA) to have scaling indices with quasiperiodic changes in complexity, as measured by scaling indices, over time. Such time series are generated by different parts of the brain, heart, and lung ONs, and the results do not depend on the underlying coherence properties of the associated time series but demonstrate a generalized synchronization of complexity. This high-order synchrony among the scaling indices of EEG (brain), ECG (heart), and respiratory time series is governed by the quantitative interdependence of the multifractal behavior of the various physiological ONs’ dynamics. This consequence of the NE opens the door for an entirely general characterization of the dynamics of complex networks in terms of complexity synchronization (CS) independently of the scientific, engineering, or technological context. CS is truly a transdisciplinary effect.

## Introduction

Since the turn of the century Network Science and Complexity Theory have been growing dramatically and their nexus has led to profoundly different ways of thinking about physiology, health, disease, and medicine in general from the modeling based on the Newtonian paradigm. The observational ubiquity of inverse power law (IPL) spectra in complex phenomena entails theory for dynamic fractal phenomena capturing their fractal dimension, dynamics, and statistics^1^. These and other properties are consequences of the complexity resulting from nonlinear dynamic networks collectively summarized for biomedical phenomena as the network effect (NE)^2^ or focused more narrowly as Network Physiology^3^. The NE is often described by homogeneous scaling variables with power law scaling having an index $\delta$ determined by the fractal dimension of the time series^4^ being a direct measure of the ON’s complexity^5^.

We adopt the network approach because the emergent macroscopic variables, whose properties are determined by their scaling indices, not by the microscopic variability, that determines the health of the body. It is the scaling of time series within a given organ that codifies its success in carrying out its function and that information is shared with the organs with which it is in contact. For example, the heart, lungs, and brain form such a triad of organs whose overall health is determined by information sharing among the ONs, as we subsequently show.

Synchronization is today identified as the mechanism needed to coordinate activities among events in any complex, multilevel, multielement dynamic network. However, as the network becomes more complex so does the changing concept of synchronization. This is particularly true of the amazingly complex ON structure of the human body and the need to coordinate activities across vastly different time scales, from the microscopic time scales of the neural networks within the brain, to the mesoscopic time scales of the cardiac and respiratory ONs, to the macroscopic time of circadian rhythms. We herein show that the complexity (scaling) of brainwave time series data is multifractal, as are the respiratory and cardiovascular time series, and that the multifractal scaling of the three are synchronous. It is likely that the two expressions, complexity synchronization (CS) and scaling synchronization, will be used interchangeably. The multifractal behavior of these time series has been identified using pairwise correlation to identify an appropriate mechanism^6^. The change in fractal scaling of time series indicates the change in complexity of the ONs as various physiological functions are performed. In the language of network science, the more complex network transfers information to the less complex network, but these roles of sender and receiver change with the functions being performed and can change in time, as well. Information is readily transported within overlapping memory areas of the heterogeneously complex brain and at any point in time a given region of the brain can receive information from sensor networks, process that information and transmit the processed signal to an appropriate physiological ON for action, depending on their function and instantaneous relative complexities^7^. This hierarchy of the average complexity is subsequently revealed by the way in which the multifractal nature of each of these three interacting ONs influence one another over time. The relative width of the multifractal spectra determines the sender (greater width) and the receiver (smaller width)^2^.

Recent advances in data processing techniques have revealed a new kind of synchronization, CS (scaling), based on crucial events (CEs)^5^, which enables the detection of synchrony among complex dynamic ONs operating on different time scales and not necessarily in stationary regimes, as we shall show. A CE time series is a renewal statistical process generated by an IPL probability density function (PDF). If the time intervals between events is $\tau$, the IPL PDF is $\psi (\tau ) \propto \tau ^{-\mu }$ and the IPL index $\mu$ is in the domain $1<\mu <3$. Asymptotically, the generated process of CEs is ergodic for $2<\mu <3$, with a finite average time interval between CEs and is non-ergodic for $1<\mu <2$, with an infinite average time interval between CEs. The scaling (complexity) of healthy brainwave time series is shown to be multifractal, as are the healthy respiratory and healthy cardiovascular time series. Moreover, the multifractal scaling of these three healthy systems is herein determined to be in synchrony with one another.

It is worth emphasizing that the operational definition of complexity adopted above is not universal but does describe a large class of phenomena having 1/*f*-variability, first observed in a physical context and named 1/*f*-noise. The power spectral density (PSD) of fluctuations is an IPL in frequency *f* of the form $S_{p} (f) \propto f^{-\beta }$ and has a long history of experiments and theories for $\beta = 1$, i.e. for 1/*f*-noise, the vast majority of which is not relevant to the problems of interest here. We confine our remarks to biomedical phenomena that are rife with time series having IPL spectra with an index in the range $0.5\le \beta \le 1.5$. Criticality is a phenomenon requiring the cooperative interaction of units, e.g., the neurons of the brain and is hypothesized as the main source of cognition. Using the criticality-induced intelligence, Mahmoodi et al.^8^ define complexity as a property of CEs, a statistical form of temporal complexity. They prove that perfect synchronization is due to the interaction between two complex networks, with the more complex network restoring the temporal complexity to the less complex network with the give and take of information over time.

### Synchronization and information exchange

The Complexity Matching Hypothesis (CMH)^5^ was a major step in understanding the nature of complexity in daily life and states that the information exchange between interacting networks is optimal when the level of complexity of the two networks are the same^9^. This information flow has also been identified with ‘strong anticipation’ and in their nomenclature the dynamics have been interpreted as ‘anticipation synchronization’ resulting from the coupling between ‘master’ and ‘slave’ networks^10^. Marmelat and Delignieres^11^ applied this interpretation to interpersonal synchronization through a matching of the complexity indices (fractal dimensions). Along with colleagues they^12^ also examined the CMH using a method based on the correlation between multifractal spectra, considering different ranges of time scales. They could distinguish between “situations underlain by genuine statistical matching, and situations where statistical matching results from local adjustments.” They analyzed empirical datasets of biannual coordination, interpersonal coordination, and walking in synchrony with a fractal metronome.

Note that the interacting networks need not be from the same scientific discipline in that network models are not discipline specific, and therefore the same model structure can be applied to networks of different origins across various disciplines (i.e., the last being a transdisciplinary phenomenon). When two complex networks interact, information flows from the more complex network to the less complex network, the more complex network perturbing the less complex network whose complexity increases until the complexity measures of the two equal ($\mu _{<} \approx \mu _{>}$) and the maximal transfer of information occurs^10,13^. Mahmoodi et al.^8^ established that scaling synchronization is a consequence of the fact that a very large number of crucial events for the IPL index in the interval $2< \mu < 3$ using the modified diffusion entropy analysis (MDEA) data processing technique, which is reviewed in due course. This notion of complexity matching has developed into the principle of complexity management (PCM)^14^ to include the influence of one network on the other when the level of complexity of the two networks are very different, which brings us to the recently developed concept of CS.

The main difference between PCM and CS is that PCM rests on adapting the linear response theory to the case where the perturbed system is characterized by an IPL index^14^, making it nonstationary, and the influence of a perturbation does not affect the IPL of the single trajectories, but rather it affects the average over infinitely many responses to the same perturbation. CS, on the contrary, is realized as a surprising synchronization between the scaling of single trajectories. This effect seems to be incompatible with the assumption that two networks interact without influencing their own complexities. In other words, networks can be initially out of scaling synchronization, and the transfer of information can directly change their level of complexity and consequently their IPL index. Experimentally, the change in complexity was observed in the rehabilitation of the elderly in the walking arm-in-arm experiment^13^. Although PCM was invoked to explain this rehabilitation effect, the model made by Mahmoodi et al.^8^ to explain this therapeutical synchronization, generated a surprising similarity between numerical and experimental results. The two interacting systems in this model change their complexity to realize the experimentally observed coordination. We believe that the CS illustrated in this paper goes beyond the limits of PCM and is closely connected to the model of Mahmoodi et al.^8^.

## Results

**Figure 1:**
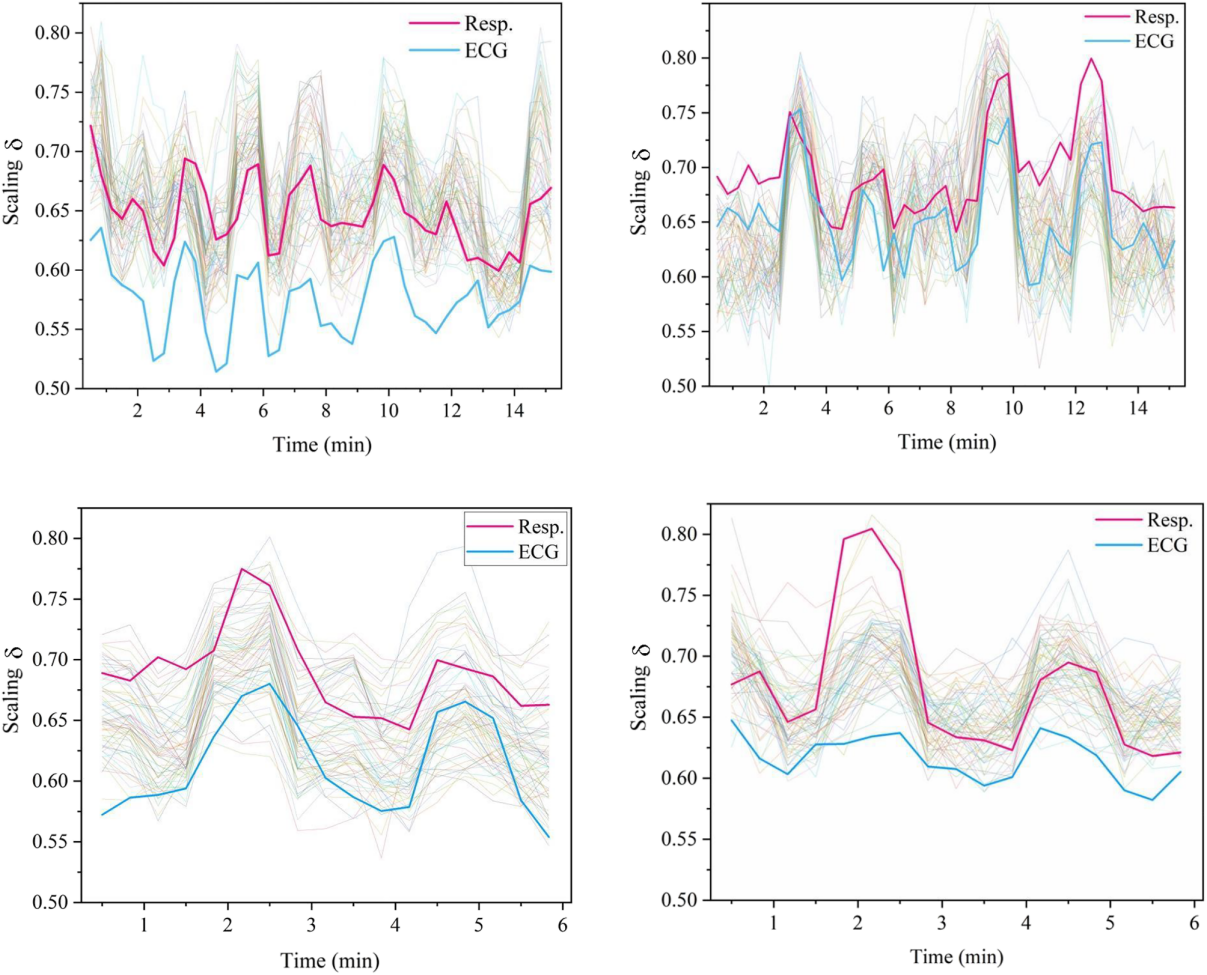
Scaling synchronization of the ONs data from participant 1 in the left column and participant 2 in the right column. Pale colored, clustered curves are the scaling over time of 64 EEG time series channels. The red and blue curves are the scaling over time of the respiration and electrocardiogram (ECG) ONs, respectively. The individual datasets were processed using MDEA (see Methods), on data windows of L = 60 s (60$\times$512 samples) duration (windows in increments of 20 s steps). The data were collected while the participants were engaged in neurofeedback training and subsequently in a Go-NoGo task (see Methods) in the upper and lower panels, respectively.

In this section we present a new way to characterize how the brain can exchange information with two other major physiological ONs, as depicted in Fig. 1, those being the respiratory and cardiovascular networks. In this figure the power law scaling indices are depicted for the processed time series, as discussed in the “Methods” section, from each of the 64 channels of a standard EEG, along with those from ECG and respiratory ONs. These 66 time series were simultaneously measured. It is clear from the figure that the quasiperiodic behavior of the scaling indices from the 64 EEG channels are in synchrony with each other as well as with those from the ECG and respiratory ONs. This figure compares the scaling results from two individuals’ time series chosen from a neurofeedback training study (see^15^ for details) based on the highest quality of raw data among all participants (i.e., the data were void of any muscle or movement artifacts) responding to two separate and distinct tasks. The upper two panels in Fig. 1 display the scaling response of the brain, lung, and heart of two individuals during neurofeedback training. The lower panel compares the response of the same two individuals during performance of a Go-NoGo task. The light curves indicate the variability in the 64 EEG channels moving in a collective way, which is a surprising result that is relatively independent of the individual and the task. The red curves show how the respiration of the lungs track the collective scaling of the brain that although differing in detail bear remarkable similarity across the two individuals and tasks. The blue curves indicate how the cardiovascular response tracks the large-scale response of the respiration process, or equally reasonable at this level of analysis, both the blue and red curves are loosely tracking an envelope function of the 64 EEG channels. What we can say with some confidence is that surprisingly, the two persons’ scale in much the same way, but the scaling differs in detail depending on the task performed and the idiosyncratic functioning of the indviduals.

The time series $X_{j} (t)$ is the output from channel *j* and scales when the time *t* is multiplied by a constant $\lambda$ resulting in; $X_{j} (\lambda t)=\lambda ^{\delta _{j}} X_{j} (t)$ and consequently, the time series is a homogeneous scaling function. Note that the scaling index $\delta _{j}$ is a local quantity in time and is related to the fractal dimension of the ‘network’ in the brain generating the time series in the vicinity of channel *j*. The time series underlying the scaling index in each of the EEG channels is seen in the figure to be multifractal with a quasiperiodic time dependence, with a similar interpretation for the cardiac and respiratory time series. Consequently, we have the time-dependent scaling index $\delta _{j}(t)$. Figure 1 provides information regarding the way the dynamics of the different parts of the brain, the heart, and the lungs influence one another. The scaling indices of all 64 EEG channels are compared with the scaling index for the cardiovascular ON (blue curve) and the scaling index of the respiratory ON (red curve). To properly interpret the behavior depicted in the figure requires that we answer the question: What is a scaling parameter and what does it entail about the underlying dynamic network?

West and Grigolini^5^ review how the IPL indices for the PDF, given by $\psi (\tau ) \propto \tau ^{-\mu }$ and for the PSD given by $S_{d} (f) \propto f^{-\beta }$ are related by $\beta =3-\mu$. The scaling index $\delta$ of the above homogeneous scaling relation determines that of the IPL index for an asymptotically ergodic time series by $\mu =1+1/\delta$ as recorded in Table 1 (An excellent review of the close relationship between the complexity of a network and the presence of fractal fluctuations (1/*f*-variability) in its macroscopic behavior is given by Deligniéres and Marmelat^16^. Consequently, either $\beta$ or $\mu$ can be used as measures of complexity by means of the scaling index $\delta$. For ergodic time series, such as that asymptotically determined by the IPL index, $\mu$ increases with decreasing scaling index $\delta$ and complexity decreases along with $\delta$. The scaling of brain activity resulting from the empirical time series considered here is seen to be typically greater than respiratory scaling which is typically greater than that of cardiac scaling. However, subsequent comparisons of the changes in scaling over time for the brain, heart, and respiratory ONs indicate that although the brain appears to have the greatest potential complexity the complexity-ordering with other physiologic ONs depends on multiple factors, e.g. health status, cognitive states, and task demands. The correlations noted here do not pertain to the underlying time series generated by the ONs, but to the scaling indices of those time series thereby being part of the new area of investigation alluded to earlier. Table 2 shows the maximum of the cross-correlation function between the time series for the average of scaling indices of the 64 EEG channels, the respiratory, and the cardiac ONs depicted in Fig. 1. Buzsaki^17^ suggests that a “small-world-like” strategy may be operative in the architecture supporting the intra-brain information transfer, see Strogatz^18^ for an excellent review of small-world theory in the present context. Buzsaki goes on to say that both theory and modeling suggest that long-range interneurons are critical for brain-wide synchronization of gamma and potentially other oscillations.

**Table 1:** This table makes easy reference to the scaling index $\delta$ from the above homogeneous scaling relation for the scaled variable *X*(*t*); relates it to the PSD $S_{p}(f)$ index $\beta$ through the waiting-time PDF $\psi (t)$ index $\mu$ in the two asymptotic regimes. The value $\mu =2$ is the boundary between the underlying process having a finite ($\mu > 2$) or an infinite ($\mu < 2$) average waiting time and is also the point at which $\beta = 1$ where the process is that of 1/*f*-noise.

	Scaled functions	Parameter relations	Parameter range	
Waiting-time PDF	$\psi (t) \propto t^{-\mu }$		1 $\leqslant$ $\mu$ $\leqslant$ 3	
Power spectrum	$S(f) \propto f^{-\beta }$	$\mu = 3 - \beta$		
Scale variable	$X(t) \propto t^{\delta }$	$\mu = 1 + \delta$	1 $\leqslant$ $\mu$ $\leqslant$ 2	Non-ergodic
		$\mu = 1 + 1/\delta$	2 $\leqslant$ $\mu$ $\leqslant$ 3	Ergodic
		$\delta = 0.5$	$\mu$ $\ge$ 3

**Table 2:** Correlation coefficients (and lower (L) and upper (U) 95% confidence intervals) among the time series of the scaling indices of the 64 EEG channels (averaged), the respiratory, and the cardiac ONs depicted in Fig. 1.

	Ave. EEG-ECG	Ave. EEG-Resp.	ECG-Resp.
Participant 1, task 1	0.6988 L 0.4802 U 0.8129	0.7255 L 0.5186 U 0.8295	0.7055 L 0.4857 U 0.8153
Participant 1, task 2	0.7401 L 0.4027 U 0.9004	0.7970 L 0.5129 U 0.9238	0.7565 L 0.4334 U 0.9073
Participant 2, task 1	0.7312 L 0.5605 U 0.8471	0.6598 L 0.4586 U 0.8032	0.7409 L 0.5693 U 08507
Participant 2, task 2	0.8722L 0.6059 U0.9457	0.8055 L 0.6731 U 0.9565	0.6803 L 0.4017 U 0.9075

Recall that the scaling index $\delta$ and complexity rise and fall together, but not in direct proportion to one another, since a phenomenon and its measure are not the same thing. One may therefore generate adjacency matrixes of scaling indices and apply graph theoretic measures to investigate dynamic network interactions as a discrete map of the changing complexity within and among regions of the brain, as well as among the three ONs, and potentially provides a new measure of the flow of information (see “Discussion” section). This suggests that we can talk about a new measure of functional connectivity, but it is perhaps premature to discuss the flow of information without having multiple time-dependent complexity maps from which to determine the changing functional role of a given channel, region, or ON.

Table 2 provides correlation coefficients (with lower and upper 95 % confidence limits) between the instantaneous average of the scaling index over all 64 EEG channels (to obtain an average scaling index for the brain), with the scaling indices for the cardiovascular ON and the respiratory ON. The correlation coefficients (all p-value < .01) are approximately the same for all three pairwise combinations in both individuals and tasks. This result suggests that in a healthy individual the complexity of the ONs is approximately the same on average, so that information flow may well satisfy the CMH on average.

## Discussion

We have defined complexity in terms of the IPL index for the waiting-time PDF for crucial events, a form of self-organized temporal complexity^19,20^ (SOTC) and shown elsewhere^8^ that complexity change results from the interaction between at least two complex networks. We have processed CE time series and determined that the IPL index is time dependent for interactions between physiologic ONs. This is distinct from the forms of coupling identified for the interaction of the same ONs studied by Bartsch and Ivanov^6^, during different stages of sleep. They^6^ determined that the interaction between the brain, cardiac, and respiratory ONs reveal pronounced phase transitions associated with detailed reorganization in network structure and links strength in response to changes in the underlying neuroautonomic regulation. Further, they identified the multifractal nature of the time series generated by the physiologic organs and identified new and interesting forms of coupling using linear coherence measures. However, lacking the data processing technique provided by MDEA, or its equivalent, they did not uncover the high-order synchrony of the time dependence of the scaling indices of the brain, cardiac, and respiratory ONs.

Deligniéres and Marmelat^16^ suggested that a network’s complexity “and the presence of 1/*f*-fluctuations in its macroscopic behavior have opened new domains of investigation”. One such domain is entailed by the multifractal nature of the scaling indices depicted in Fig. 1. The multifractality indicates that all three (different parts of the brain, heart, and lungs) ONs have dramatic changes in complexity over time, as indicated by the quasi-periodic time dependence of the scaling index, being a direct consequence of their inter- and intra-ON interactions. There is no set hierarchy among the scaling indices for parts of the brain and the physiologic ONs they monitor and with which they exchange information. The brain sends and receives information from various sensor ONs. For example, on 9/11 people saw smoke billowing from the two towers; as the towers collapsed, they smelled the heat and choked on the clouds of dust and debris; they heard the screams of those not realizing what was happening to them. The brain processes the sensor signals and from the synthesis of the processed information, makes an executive decision of either ‘flight or fight’, ultimately transmitting the decision for action to the motor control network. The average person fled from the site, while the first responders fought their way into the devastation. The drama of that day highlights not only that the human brain receives information from the five senses of sight, sound, smell, taste, and touch, but subsequently transmits information to motor control and physiological networks in response to its processing of the information received. In the final analysis, the brain determines whether you make it to safety or have your name inscribed on a remembrance granite slab.

Note that the scaling indices are changing over time in Fig. 1, thereby indicating the multifractal nature of the time series from the brain, cardiac, and respiratory ONs. This characterization of the dynamics suggests a parallel with Huygens’ observations of the “sympathy of two clocks” in that his observation lacked the theoretical foundation subsequently provided by Newton’s mechanics, and we also lack a comprehensive theory with which to explain the necessity for this manner of controlling physiologic signals. However, unlike Huygens we have the advantage of a three-century perspective of how to use the scientific method to develop theory necessary to further understanding. In this regard we call attention to the neuroscientist Buzsaki^21^, who observed that transient coupling between various parts of the brain may support information transfer as described by ‘small world theory’ and the scaling results shown in the figure support this conjecture. But a word of caution is appropriate here because the synchrony observed in the scaling indices is not directly related to the synchronous behavior observed from the correlation and other central moment properties of the time series.

We established the connection between multifractality and CEs in surrogate time series^7^. Elsewhere^22^, we found the same connection in physiologic time series using heartbeat data, thereby supporting the fundamental role that CEs play in the exchange of information between interacting complex ONs. Therein we established the consistency of two apparently different diagnostic techniques. The first technique is based on the multifractal spectrum of healthy individuals being broader than that of pathologic subjects. The second technique is based on heartbeat dynamics being a mixture of CEs and non-CEs time series, with pathologic patients having a high probability of hosting a greater percentage of non-CEs. Moreover, we proved therein that increasing the fraction of non-CEs in the heartbeat time series reduces the width of the multifractal spectrum thereby simultaneously establishing compatibility of the two techniques, while providing a dynamic interpretation for the source of multifractality as being due to the level of complexity of the ON, using the 1/*f*-variability scaling index as the complexity measure.

It also needs to be pointed out that there is a distinct difference between chaos synchronization and CS. A single nonlinear dynamic network in the process of chaos synchronization is chaotic and surprisingly two such networks can synchronize while simultaneously maintaining the chaotic dynamics they had in isolation. The theoretical question that needs to be answered has to do with the generalizability of the underlying theory, which is a substantial part of the immediate direction of our future research. The analysis of the three heterogeneous neurophysiological data sources clearly indicates from the fractal structure of each that the scaling of the underlying neural networks, cardiovascular and respiratory networks are all tightly coupled, but in a way that is not obvious, since we do not use the time series to construct any kind of correlation function. Given that the fractal dimension is related to the scaling index for each dataset we conjectured that the width of the fractal distribution for the brain would be the widest when the brain is the healthiest. One way to support that conjecture would be to answer the open research question: How can we analyze the multifractality for relatively short time scaling series for a single subject?

## Conclusion

A major result of the present work is that we have shown that when the scaling parameter $\delta$ is greater than 0.5 there is a fork in the road beyond $\delta$ = 0.5 leading to two distinctly different understandings of the dynamics of the complex network being considered. The time series generated by an ON beyond the fork would have two very different kinds of statistics depending on which branch is found from the data. The branch of one leg beyond the fork is interpreted by Mandelbrot to be fractional Brownian motion (FBM)^23^, which has Gaussian statistics and an infinite memory. Whereas the other branch of the fork has the statistics interpreted by the Grigolini-West group^5^ to be CEs, in which the statistical fluctuations are renewal, and the PDF is given by the scaling form of Eq. (1) with a quite general non-Gaussian form. It is this second branch that describes the dynamics of healthy complex ONs. It is not just that other investigators assume that ONs have statistical properties described by FBM^6,11–13,16,23^, which they do, but that none of them to the best of our knowledge, even tip their hat to the possible existence of the alternative CE branch. The results of the present paper establish that broad multifractal spectra transfer information between complex ONs by means of CEs. This is consistent with earlier findings of our group regarding the connection between the multifractal spectrum and SOTC fluctuations^8^ and subsequent findings^5,7,24^ all supporting the surprising conclusion reached in the above paragraph. Consequently, this alternative interpretation of the statistics fulfills the promise made by Ivanov et al.^25^ of the new fields of network physiology and network medicine giving rise to significant progress in understanding biomedical phenomena. In summary, we list the theoretical findings supported by the MDEA of the time series generated by the interacting brain, heart, and lung ONs:

1. The time series generated by an ON *X*(*t*) is a homogeneous random fractal having a self-similar scaling index $\delta$, whose fractal dimension $D = 2 - \delta$ is a direct measure of the complexity of the time series.
2. The scaling index is typically time-dependent $\delta (t)$ capturing the multifractality of the ON time series through the time dependence of the fractal dimension.
3. CS is the mechanism necessary to coordinate the time-dependent scaling behavior of the time series for interacting ONs.
4. It is worth speculating that the techniques developed in network science, particularly those in multilevel ONs, may lead to a significantly deeper understanding of connective functionality of the brain than has been provided by less robust modeling techniques.

Although consistent with CS, we cannot say definitively from the data presented here (two subjects performing two different tasks) when one network is sending and the other receiving information. Future research is needed to determine which factors may influence the sender/receiver hierarchy.

## Methods

### Participants

Two participants with the cleanest (artifact-free) raw data were selected from a neurofeedback training study^15^ for analysis and the results are presented in Fig. 1 and Table 2. Volunteers who agreed to participate were asked to read and sign an Informed Consent Agreement (approved by the Human Use Committee at the US Army Research Laboratory and the Institutional Review Board at the University of Maryland, Baltimore County, in accordance with the Declaration of Helsinki and the U.S. Code of Federal Regulations).

### Signal acquisition and processing

All data were acquired simultaneously at 2048 Hz and referenced online to the Common Mode Sense (CMS) and Direct Right Leg (DRL) electrodes using a 64 (+8 external) channel BioSemi system (Amsterdam, The Netherlands; http://www.biosemi.com/products.htm). An integrated auxiliary channel was used to acquire respiration data (Nihon Kohden TR-753T respiration belt). Signal processing of electroencephalographic (EEG), electrocardiographic (ECG), and respiration (RESP) time series was applied using EEGLAB (ver 14.1.2b; http://sccn.ucsd.edu/eeglab/) and in-house code using MATLAB (9.3.0.713579; Natick, MA) on a 64-bit Linux operating system. EEG and ECG data were high pass filtered at 1 Hz and RESP data were low pass filtered at 2 Hz using zero-phase finite impulse response filters. EEG data were re-referenced to the average of all 64 EEG channels, down sampled to 512 Hz and cleaned of artifacts using independent component analysis^26^. ECG and RESP data were also down sampled to 512 Hz after filtering.

### Theory

We now answer the question posed earlier: What is a scaling parameter and what does it entail about the underlying dynamic network? For a stochastic process the dynamic variable *X*(*t*) with homogeneous scaling is given by $X(\lambda t) = \lambda ^\delta X(t)$ and more generally is interpreted in terms of the scaling PDF:

1 $$P(x,t)=1/t^{\delta} F(x/t^{\delta}),$$

where *P*(*x*, *t*)*dx* is the probability that the random dynamic variable *X*(*t*) is in the interval ($x, x + dx$) at time *t*. The PDF function *F*(.) is unknown in general, however for $\delta$ = 0.5 this PDF is Gaussian, and the process is diffusive. If the PDF is Gaussian but $\delta$ is not equal to 0.5 the process is said to describe fractional Brownian motion (FBM), as first described using the fractional-order calculus by Mandelbrot and van Ness^1^. The more interesting case is when the unknown function is not Gaussian, for example, when the process is Lévy stable. Using the definition of Shannon/Wiener entropy we obtain using the scaling PDF, without knowing the *F*(.) function, the deviation of the entropy from its reference state defined by the unknown function is:

2 $$\Delta S(t)=S(t)-S_{ref} = \delta ln(t).$$

**Figure 2:**
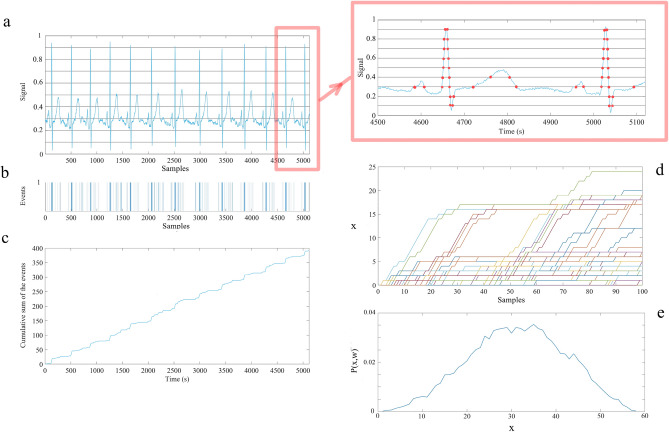
The schematic of the Modified Diffusion Entropy Analysis (MDEA). Panel (**a**): The blue curve is L = 10 s ($10\times 512$ samples) of the heart rate signal (as an example time series) which was first projected to the interval [0,1] and then divided into ten stripes of size of 0.1. The horizontal lines in panel (**a**) define the stripes. The top panel on the right is a zoomed in section of the panel (**a**) where the red circles represent the events as the times when the signal (blue curve) passes from one stripe to another. Panel (**b**): The extracted events in panel (**a**) assigned the value 1 to create a binarized time series. Panel (**c**): The diffusion trajectory made by cumulative summation of the events of panel (**b**). Panel (**d**): Slices (of length $w =100$ samples; 100/512 samples/s = 0.195 s and starting from an event) from the diffusion trajectory which are shifted to start from the origin. Panel (**e**): The histogram of the position of the trajectories of panel (**d**) at the end of the window ($w=100$ samples). To create the histogram of panel (**e**) we used L = 60 s ($60\times 512$ samples) of data and stripe size of 0.01, which are the MDEA parameters used in this work.) Note that for purposes of illustration the above figures (panels (**a**)–(**d**)) are only L = 10 s (10$\times$512 samples) of data and we chose stripe size of 0.1 to enable visualization of individual heart beats.

In this case t refers to the variable temporal window ‘w’ (see “MDEA” section). Consequently, a graph of the entropy for such a process versus the logarithm of the time is a straight line whose positive slope gives the scaling index. The time derivative of the entropy (information) given by Eq. (2) asymptotically vanishes as an IPL with unit index, entailing that the average of the stochastic variable asymptotically becomes constant. Diffusion entropy analysis (DEA) was originally introduced to study the complexity of a social process^27–29^. There is a significant difference between the DEA and its generalization to modified DEA (MDEA). The DEA records a positive unit step in the diffusion trajectory for every event in the dataset whether it is a CE or not. Many of these diffusion trajectories for a given dataset are collected into an ensemble, the ensemble PDF is calculated from the histogram and subsequently inserted into the expression for the Shannon/Wiener entropy. The resulting time-dependent entropy is plotted against the logarithm of time and yields the scaling index $\delta$ as predicted by Eq. (2). The fact that the empirical time series contains both CEs and non-CEs is a limitation of the DEA technique, which is resolved by the generalization to MDEA. The modification is to partition the y-axis into many equal-sized intervals (stripes) and record each crossing of the time series from one stripe to a different stripe as an event (see Fig. 2) and construct the new diffusive trajectory by accumulative sum of the events. This use of stripes filters out the influence of the non-CEs in the empirical time series on the diffusion trajectory, resulting in a more accurate measure of the scaling index over a longer time than obtained using DEA.

## Modified diffusion entropy analysis (MDEA)

MDEA was applied to post-processed continuous data from all 64 EEG channels, the ECG, and the RESP time series of two participants in one session of neurofeedback training and Go-NoGo task. Estimates of scaling indices were obtained on every L = 60 s ($60\times 512$ samples) data slice in increments of 20 s steps, yielding a time series of scaling exponents for each channel. For MDEA of each slice of the datasets it was first projected onto the interval [0,1] (i.e., the time series subtracted from its minimum and the resulting time series divided by its maximum, thereby normalizing each of the three different time series) which was then divided into parallel stripes of size 0.01 ( note in panel (a) of Fig. 2, ECG data plotted and stripe size of 0.1 used for visualization purposes). Next, the events were extracted as crossings of the signal from one stripe to another (top-right panel of Fig. 2) and each marked as 1 denoting a unit step in the diffusion process (panel (b) of Fig. 2). Using the extracted events, we created a diffusion trajectory (panel (c) of Fig. 2 i.e., the cumulative sum of the events in panel (b)).

To determine the statistics of the single diffusion trajectory (blue curve in panel (c) of Fig. 2), we select a window size *w* and slice the diffusion trajectory into many pieces, each of them starting from an event. By shifting all the slices to start from the origin (panel (d)), we evaluate the distribution of trajectories (histogram) at time *w* (panel (e)). Using these distributions for different window sizes *w* we can define the Shannon/Wiener Entropy for the underlying process, assuming that *P*(*x*, *w*) is the PDF corresponding to window size *w*, to be:

3 $$S(w) = -\int P(x,w)lnP(x,w)dx.$$

**Figure 3:**
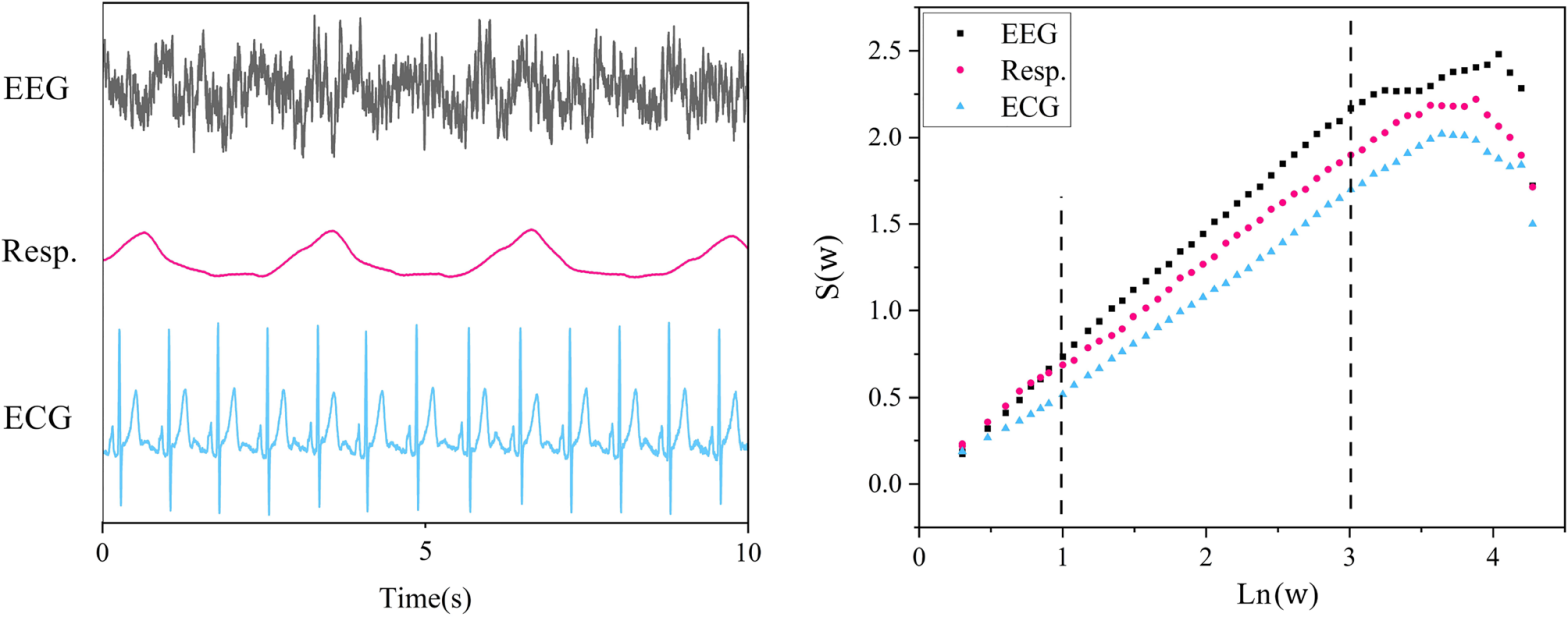
Left panel: L = 10 s (10$\times$512 samples) of a single channel of EEG, respiration, and ECG data. Right panel: The corresponding MDEA graphs of the time series on the left panel (for L = 60 s; $60\times 512$ samples). The slopes of the linear parts are the measure of temporal complexity of the time series. Note, for illustration purposes, we show 10 s time series in the left panel, and on the right panel for the same EEG channel, ECG, and respiration time series we show the scaling indices computed over a 60 s window.

If the PDF is given by Eq. (1) with *t* replaced with *w*, we obtain Eq. (2) with the same replacement. So, the slope of $\Delta S(w)$ vs. *lnw* will give the scaling index $\delta$, as shown in Fig. 3 for one slice (L = 60 s; 60$\times$512 samples) of data of the three different physiologic signals. After evaluating the scaling index for each of the time series for 64 EEG channels, the EEG, and the respiratory ON datasets, using MDEA, we studied the cross-correlation between them. The results confirm the strong connection between the complexity of the three ONs depicted in Fig. 1.

The change in the count of CE in any given time interval is a projection of the very different shapes of continuous time series, as depicted in Figs. 2 and 3, onto temporal statistics. A feature with a long gradual slope translates into a few positive random walk (RW) steps which are well-spaced in time. On the other hand, a feature with a steep short sloping curve transforms into many closely spaced positive RW steps, the steeper the empirical curve the denser the number of steps taken in the temporal representation. This is made evident by the inserted modification in Fig. 2a. This is the reason for normalizing each dataset rather than using the raw empirical times series to compare statistics. Normalization provides a common interval upon which an empirical feature can be transformed onto its temporal statistics and the statistics are those of the independent fraction of the total time interval covered between each event.

This procedure of normalizing the total duration of the diffusion trajectory and projecting a feature of the continuous time series onto the normalized interval eliminates an illusion created by the eye in examining the empirical features of the continuous time series. When the span of the space scales in continuous time series significantly differ from one time series to another, say that of a typical EEG channel trace to that of a simultaneous ECG, the visual comparison tricks us into thinking that the two traces are so different that any significant interaction between them must be manifest in a certain way. For example, the two-point correlation function must reveal any hidden interactions. But this is a bias we have that need not be realized for truly complex continuous time series. It certainly is not the case for the triad of ONs time series considered here. The interaction occurs among the fractal dimensions of the three interacting ONs and may or may not show changes in the two-point cross-correlation functions.

### Take-away messages concerning CS

1. The scaling behavior of these three physiologic time series is invisible to most data processing techniques and thereby so too are the CE.
2. The hidden interdependence is above the level of time series scaling generated by the interactions of the three ONs.
3. It is only after MDEA processing that the CS mechanism tying the three ONs together is revealed.
4. CS is a newly identified evolutionary mechanism devised by mother nature to enable a network-of-ONs to continue performing its global functioning even within a complex dynamic host environment.
5. The multifractal dimension indicates how information is encoded within ON time series.
6. The time-dependent fractal dimensional encoding insures efficient communication across multiple interacting ONs.
7. The quasi-periodic oscillations are each statistically disrupted by distinct IPL temporal frequency perturbations.

## Data Availability

The MDEA Matlab code is available at https://github.com/Korosh137/MDEA.git. All data are available upon request (K.M. or S.E.K.)
